# The Courts and Erie County Medical Society

**Published:** 1863-09

**Authors:** 


### EDITORIAL DEPARTMENT.

### THE COURTS AND ERIE COUNTY MEDICAL SOCIETY.

The County Medical Societies are branches of the New York State Medical Society, and have been regarded in former years as essential to the interests of the regular profession. At the time of organization, they were made by law a protection to the practice of legitimate medicine, but have long since by statute ceased to possess any such function. While it has been obvious that the organization was of no practical value, it has been sustained upon the ground of forming a medium of connection with the State Society, and constituting a sort of dividing line between legitimate and irregular practice. It was supposed that the Society could regulate in some degree its own affairs, could at least select its fellows and protect itself from what was regarded as improper and unworthy membership.

By the following section of an Act passed April 10, 1813, it appears that by-laws could be legally made regulating the admission and expulsion of members; the Society have always acted under this impression, and have formerly been sustained in their action:

"*And be it further enacted,* That it shall be lawful for the respective Societies to make such by-laws and regulations relative to the affairs, concerns and property of said Societies, relative to the admission and expulsion of members, relative to such donations or contributions as they or a majority of the members at their annual meeting shall think fit and proper: *Provided,* That such by-laws, rules and regulations made by the Society of the State of New York, be not contrary to, nor inconsistent with the Constitution and laws of this State, or of the United States; and that the by-laws, rules and regulations of the respective County Societies shall not be repugnant to the by-laws, rules and regulations of the Medical Society of the State of New York, nor contrary to, nor inconsistent with the Constitution and laws of this State or of the United States."

So far as we are informed the right and duty of the Society to guard itself from improper and dishonorable membership was not questioned until within the last few years, when appeal has been made to the Courts upon several occasions for admission by those who had been refused its fellowship. The virtual decision of the Court, has been to the effect, that the physicians belonging to the Erie County Medical Society, have no legal right to select their companions; that the Courts have the right to admit and retain physicians in this organization, or to expel when their conduct is judged by them sufficiently dishonorable and unprofessional to warrant such action. This Society, if a purely legal association, at present affords no legal protection whatever; the graduate of a respectable Medical College has all the rights and privileges which pertain to members of the County or State Societies, and there is no longer the slightest benefit to be derived from these organizations so far as professional assistance, or legal protection is concerned. That the Courts should presume to decide who are, and who are not, suitable persons for membership in our Medical Societies, is one of the most absurd propositions ever presented, and submission to such dictation would prove the Societies incapable of self-government and unworthy the trusts confided to them.

We have been deluded in the view that our State and County Societies were honorable associations, for the protection and promotion of medical science, for it appears at length that graduates of medical colleges may practice the grossest empiricism, make the most public exposure of their charlatanism, obtain the favor and support of the easily fooled, and at length after the pretention has grown old, and the rewards of empiricism and mountebankry more precarious and uncertain, may assume the "*locus penetentiæ*," and be regarded by the Courts as entitled to all the rights and privileges of good fellowship; that they may do this and yet never make any public acknowledgment of their errors, placing so far as the masses are concerned, the advocate and advertiser of an exploded and obsolete system of pretention, upon an equality with the members of an honorable profession. It looks in making a truthful picture as if we had some actual life scene to draw from, and were growing personal in our allusions to the Courts, and to the members admitted through them to the Society. This is not so at all. We do not mean any such thing, It is the principle we oppose, and nothing more. If the Courts admit our members as they judge proper, and retain them as they see best, offering acceptance with all sins unrepented and unforgiven, at least unacknowledged only at the confessional, where the curtain of secrecy is closely drawn, then at our next meeting ws expect the Court will admit the few

Homeopathists in the City who hold diplomas from medical colleges, for it is now pretty well settled that they occupy the "*locus penetentiœ*" as truly and as heartily as any other, while in some cases their actual practice is sufficiently orthodox for the Judges and Courts, "and surely the door of repentance is not shut against them, and they may avail themselves of the right and duty to reform."

The Courts are opening the door of admission to our County Society so widely and protecting it so feebly that if it ever was of any practical benefit to the profession, it is now wholly destroyed and the Society is made only the medium whereby quackery may obtain better company. To the regular physician it has no value. Indeed it is astonishing how even without this finishing touch of destruction, physicians could so long have respected and sustained this organization, yearly going through the miserable farce of electing officers, collecting dues, discussing the by-laws, etc., etc., when in point of fact the soul and spirit of the organization had long since taken its departure, leaving only the recollection of its former worth in the minds of a few of the "oldest inhabitants." Pity that its skeleton form, could not have gone with the pure spirit which once inhabited it, and rested in a common and honored grave.

It may be difficult to make satisfactory disposition of this ghost, but present or absent, sustained or repudiated, regarded or neglected, it is only the shadow without the substance. The Erie County Medical Society protects no interest, redresses no wrong, cultivates no medical or other science, makes now no dividing line between legitimate, honest and honorable medical practice, and the grossest forms of pretention and quackery. It is capable of evil, but wholly incompetent of good. But why speak the truth so plainly of an institution which has been so long respected? Why not let the dead rest in peace? We are law loving, law abiding citizens, and do not propose any conflict with the institutions of law. We do however propose for the consideration of the members of Erie County Medical Society, the abandonment of an organization which does them no good, and appears now likely to do them great harm. A Medical Society whose membership is under the control of our Courts and Judges, which proposes no advancement of medical science, and offers no protection to medical practice, is a nuisance and a humbug; and sinks into insignificance when standing by the side of a voluntary association whose chief object is the cultivation and promotion of medical and surgical knowledge, whose membership is thus far above suspicion, and wholly under its control, in no way liable to dictation or interference.

The County Society once abandoned, dead and buried, its resurrection is impossible with its present provisions. The State Society has kept up a nearer semblance of life, and as forming a connection with it, the County organization may still have its advocates. This is the only conceivable argument in its favor, and we are willing it should have its full influence since for this loss of communication we have a remedy to propose which may be made effectual, but if there is no remedy, it is well, of two evils to choose the least.

We have for a number of years been trying to sustain the integrity of this Society, both in the Courts and elsewhere. It has cost us as a profession a great expenditure of kind feeling, and has been productive of no good whatever. It has seemed to augment and make public the differences among medical men, while it has never conferred one single advantage upon either party in the contest. Looking at the interests of any physicians who have been admitted to membership by force of legal process, it has never accomplished for them any good; it is impossible by this process to obtain honorable and cordial fellowship, it is in reality no membership at all,

> "You can *call* spirits from the vasty deep, but,
> Will they come when you do call them?
> That isthe question."

The Courts can order membership in Medical Societies, but, will it come when they do order it? that is the question.

Whoever desires to defend this Society will please send his copy.— We have advocated its demise, and briefly indicated our reasons.

